# Arginase 2 deficiency mitigates sepsis‐associated acute kidney injury by alleviating lipid accumulation

**DOI:** 10.1002/ctm2.70770

**Published:** 2026-09-18

**Authors:** Yu Xin, Yanqi Liu, Ning Zhang, Pengfei Huang, Qianqian Zhang, Yinghao Luo, Xinran Wang, Xiuhua Zhang, Tao Wu, Fengye Liu, Yu Xiao, Lifeng Shen, Weiting Zhang, Xibo Wang, Xianglin Meng, Yuping Bai, Xuan Wang, Nan Zhang, Qianghong Xu, Hongxue Meng, Xubin Zheng, Lixin Cheng, Kaijiang Yu, Changsong Wang

**Affiliations:** ^1^ Department of Critical Care Medicine the First Affiliated Hospital of Harbin Medical University Harbin Heilongjiang Province China; ^2^ Central Laboratory of The First Affiliated Hospital of Harbin Medical University Harbin Heilongjiang Province China; ^3^ Heilongjiang provincial key laboratory of critical care medicine Harbin Heilongjiang Province China; ^4^ Department of Critical Care Medicine Shenzhen People's Hospital (The First Affiliated Hospital of Southern University of Science and Technology) Shenzhen China; ^5^ Department of Geriatrics Guangdong Provincial Clinical Research Center for Geriatrics Shenzhen Clinical Research Center for Geriatrics Shenzhen People's Hospital (The First Affiliated Hospital of Southern University of Science and Technology) Shenzhen China; ^6^ Health Data Science Center Shenzhen People's Hospital Shenzhen China; ^7^ Department of Critical Care Medicine Zhejiang Hospital Hangzhou Zhejiang China; ^8^ Department of pathology Harbin Medical University Cancer Hospital Harbin Heilongjiang Province China; ^9^ School of Computing and Information Technology Great Bay University Guangdong China; ^10^ Dongguan Key Laboratory for AI and Dynamical Systems Guangdong China

**Keywords:** arginase 2, L‐arginine, sepsis‐associated acute kidney injury, spatial multi‐omics

## Abstract

**Background:**

Sepsis‐associated acute kidney injury (S‐AKI) imposes substantial morbidity and mortality burdens in critically ill populations. This study aimed to construct a spatiotemporal multi‐omics atlas of kidneys in a mouse model of S‐AKI using spatial metabolomics and proteomics, and to explore potential therapeutic targets for S‐AKI.

**Methods:**

A spatiotemporal multi‐omics atlas of S‐AKI mouse kidneys was constructed using spatial metabolomics and proteomics. Arginine metabolism was evaluated via L‐arginine supplementation. ARG2 was inhibited by nor‐NOHA or renal tubule‐specific knockdown. Kidney injury was assessed by histopathology, real‐time glomerular filtration rate (RT‐GFR), serum serum urea nitrogen (BUN), and injury markers. RNA‐seq and serum metabolomics elucidated nor‐NOHA's mechanism, with in vivo verification of pathway activation and lipid reduction. In vitro studies used ARG2 KO renal primary tubular epithelial cells (RPTCs) and kidney organoids.

**Results:**

Arginine metabolism played a crucial role, but L‐arginine supplementation did not improve renal function. ARG2 was significantly upregulated in proximal and distal tubules of the renal outer medulla and in macrophages. Treatment with the arginase inhibitor nor‐NOHA or knockdown of ARG2 in renal tubules significantly alleviated kidney injury, as evidenced by reduced tubular injury, increased RT‐GFR, decreased BUN, and reduced injury markers. RNA‐seq and serum metabolomics showed that nor‐NOHA significantly reduced lipid accumulation in renal tubules and serum, with significant enrichment of the peroxisome proliferator‐activated receptor (PPAR) pathway centered around PPARγ. In vivo experiments confirmed pathway activation and alleviation of lipid accumulation. In vitro experiments using RPTCs from ARG2 knockout mice and human kidney organoid model revealed that ARG2 knockout restored PPARγ expression and reduced tubular lipid accumulation.

**Conclusions:**

Inhibiting ARG2 in renal tubules during S‐AKI may alleviate lipid accumulation and kidney injury by activating PPARγ, providing a new therapeutic strategy.

**Key points:**

- Spatiotemporal multi‐omics analysis constructed a detailed atlas of S‐AKI kidneys, revealing the regional specificity of metabolic and protein expression in different kidney regions during the progression from sepsis to S‐AKI.
- Arginine metabolism plays a key role in the pathogenesis of S‐AKI, with arginine levels continuously decreasing in S‐AKI and significantly correlating with renal function.
- In S‐AKI, ARG2 expression increases, mainly localizing to renal tubular cells and macrophages. Renal tubule—specific ARG2 knockdown reduces renal tubular lipid accumulation and kidney injury in S‐AKI, likely playing an important role by regulating PPARγ to affect lipid accumulation.
- The kidney organoids with ARG2 knockout further confirmed the key role of ARG2 in S‐AKI, providing a new therapeutic target for S‐AKI treatment.

## Graphical abstract

1. Spatiotemporal multi‐omics atlas reveals regional metabolic and proteomic specificity during sepsis‐to‐S‐AKI progression.

2. Arginine metabolism is central to S‐AKI pathogenesis, with declining arginine levels correlating with worsening renal function.

3. ARG2 upregulation in renal tubules and macrophages drives tubular lipid accumulation and kidney injury via PPARγ suppression.

4. Genetic and pharmacological ARG2 inhibition, validated in mice, primary cells, and human kidney organoids, restores PPARγ activity, reduces lipid deposition, and alleviates S‐AKI, identifying ARG2 as a promising therapeutic target.

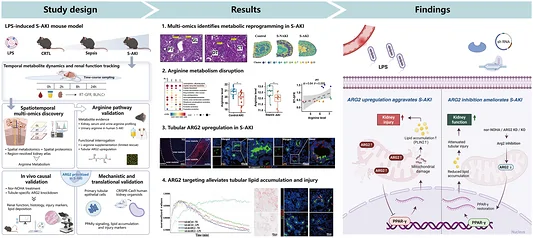

## 1 INTRODUCTION

Critically ill patients with acute kidney injury (AKI) face markedly elevated mortality and morbidity risks.^1^ As the most common cause of AKI, sepsis accounts for approximately 6 million cases of sepsis‐associated acute kidney injury (S‐AKI) worldwide annually.^2^ End‐stage renal dysfunction caused by poor prognosis brings heavy burden to patients and society.^3^ The mechanism of S‐AKI is complex, although there are many related mechanisms, such as metabolic reprogramming, immune inflammatory response, microcirculation dysfunction, etc., there is still a lack of specific targeted therapy.^4^, ^5^, ^6^

The pathogenesis of AKI is characterized by an intricate interplay of cellular and molecular mechanisms that ultimately dictate the fate of the kidney, determining whether it will fully recover or suffer irreversible damage. Given this complexity, spatial metabolomics and spatial proteomics technologies are indispensable. They offer unprecedented insights into the spatial distribution and dynamic interactions of metabolites and proteins within the kidney, enabling a more nuanced understanding of the pathophysiological processes at play in S‐AKI.

Defects in fatty acid β‐oxidation (FAO) play a crucial role in renal injury. Lipid accumulation in renal proximal tubular epithelial cells (RPTCs) provokes lipotoxicity, manifesting as mitochondrial impairment and elevated reactive oxygen species (ROS) generation, loss of adenosine triphosphate (ATP) production, apoptosis and elevation of inflammatory cytokines, ultimately exacerbating renal damage.^7^, ^8^ The peroxisome proliferator activated receptor (PPAR) pathway, particularly peroxisome proliferator‐activated receptor gamma (PPARγ), has garnered increasing attention in renal diseases due to its key role in regulating lipid metabolism, anti‐inflammation and cell protection.^9^ However, the upstream mechanisms controlling FAO disruption in RPTCs remain unclear.

Arginine metabolism plays a pivotal role in the pathogenesis and progression of S‐AKI, contributing to renal tubular epithelial cell injury and repair through multiple mechanisms, including modulation of nitric oxide synthesis, urea cycle function and immune‐inflammatory responses. Its catabolic enzyme arginase 2 (ARG2), a kidney mitochondrial protein, is aberrantly upregulated in acute kidney injury (AKI) induced by diverse aetiologies. However, whether ARG2 acts as a pathogenic factor or a protective/therapeutic agent in acute kidney injury remains controversial.^10^, ^11^, ^12^, ^13^, ^14^, ^15^ Studies have shown that ARG2 plays a crucial role in high‐fat/high‐sucrose diet‐induced inflammation and metabolic dysfunction in visceral adipose tissue (VAT), and its deficiency alleviates lipid accumulation and inflammatory response in adipocytes by upregulating PPARγ and related lipid metabolism genes.^16^ In an obese mouse model, the expression and activity of renal ARG2 are enhanced, which is associated with increased renal lipid accumulation and oxidative stress. However, these changes can be alleviated by ARG2 gene knockout, indicating that ARG2 plays an important role in obesity‐related kidney damage.^17^ However, the role of ARG2 in S‐AKI and its relationship with lipid metabolism remain unclear.

In this study, we first constructed a multi‐omics landscape of S‐AKI kidneys through spatiotemporal multi omics analysis. We identified that the arginine metabolism plays a crucial role. Subsequently, using mice, RPTCs and kidney organoids to establish in vivo and in vitro models, we mapped ARG2 expression to specific renal cell types during S‐AKI, elucidated its underlying pathogenic mechanisms, and explored its potential as a therapeutic target.

## 2 RESULTS

### 2.1 Construction of a Spatiotemporal Multi‐omics Atlas of Sepsis‐induced Acute Kidney Injury

**FIGURE 1:**
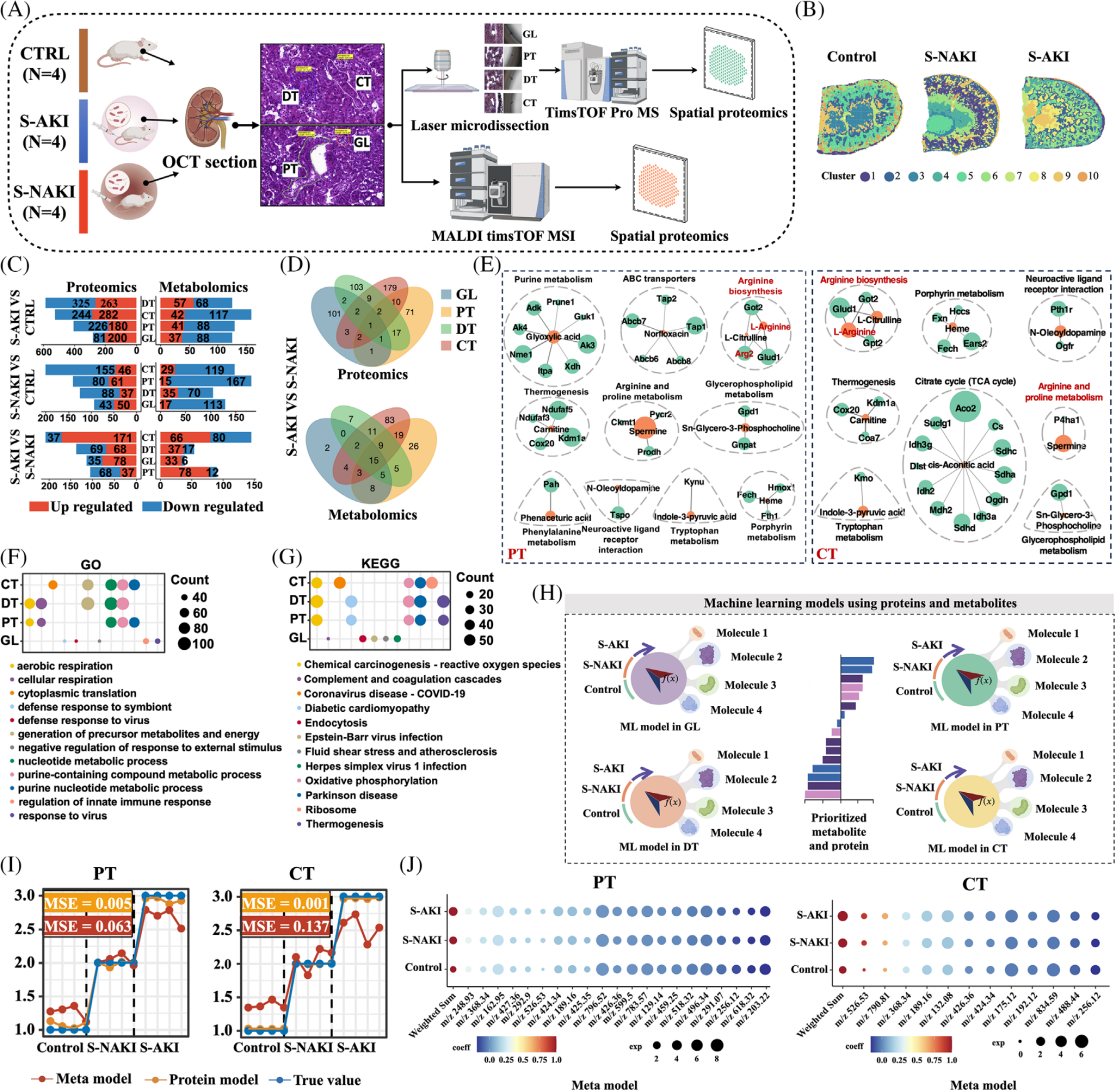
Spatial multi‑omics reveals kidney metabolic heterogeneity between sepsis and S‐AKI. Experimental workflow. The study established three mice models: Control, Sepsis and S‐AKI (*n *= 4 per group). Kidney tissue sections from each model were subjected to concurrent spatial metabolomic and proteomic profiling. (B) Spatial segmentation map of kidney tissues. The map was generated by spatially‐aware nearest shrunken centroids clustering, which incorporates a spatial weight matrix into the classic nearest shrunken centroids algorithm to integrate the metabolic profiles of neighbouring pixels and preserve spatial continuity during segmentation. This map provided a reference for selecting renal compartments in subsequent analyses. (C) Bar plot showing the number of DEPs and DEMs across distinct renal compartments. (D) Venn diagrams illustrating the overlaps of DEPs and DEMs among different renal compartments. *p*‐values were calculated by hypergeometric test (Fisher's exact test) for each pairwise comparison. All pairwise overlaps were significantly lower than expected by chance (*p* < 0.05), supporting the functional heterogeneity among compartments. (E) Integrated pathway network depicts KEGG pathways that were significantly co‑enriched by both the DEPs and DEMs. (F) Gene Ontology functional enrichment analysis of the DEPs. (G) KEGG signalling pathways enriched with DEPs. (H) The diagram illustrates the workflow for building machine learning models that selected protein and metabolite biomarkers to classify three phenotypes. (I) Performance comparison between the proteomic and metabolomic models. (J) The bubble plot integrating metabolite expression and model coefficients, with bubble size indicates relative expression level and colour represents the model coefficient. CT, Collecting ducts; DT, Distal tubules; GL, Glomeruli; PT, Proximal tubules.

To systematically construct the spatiotemporal molecular landscape of S‐AKI, we established mice models representing three distinct stages: Control (CTRL) (*n* = 4), Sepsis (S‐NAKI) (*n* = 4), and Sepsis‐induced AKI (S‐AKI) (*n* = 4). Kidney tissues were subjected to high‐throughput spatial metabolomics and spatial proteomics profiling (Figure 1A). In total, we identified 1042 metabolites and 4524 proteins with spatial resolution. Using the spatial metabolomics data, we performed spatially‐aware nearest shrunken centroids clustering (See Supporting Information Methods for a detailed description of the algorithm), delineated the inherent metabolic heterogeneity between the inner and outer medulla based on the global metabolic profiles (Figure 1B). Combined with pathological H&E images, we annotated four renal compartments, including the glomeruli, proximal tubules, distal tubules and collecting ducts, to investigate the heterogeneity across distinct renal regions during the progression from sepsis to S‐AKI.

To elucidate the pathophysiological progression from sepsis to S‐AKI, we performed integrated metabolomic and proteomic analyses across multiple regions of interest (ROIs) in the kidney. Differential expression analysis revealed that the most prominent injury‐related changes in S‐AKI were concentrated in the collecting duct, where the number of differentially expressed metabolites (DEMs) and proteins (DEPs) substantially exceeded that of most other regions, followed by the distal tubule and proximal tubule (Figure 1C). This pattern likely results from the collecting duct's unique position as the terminal segment of the nephron. It accumulates metabolic byproducts from upstream injury, undergoes its own metabolic alterations, and is exposed to highly concentrated urine, resulting in the greatest diversity and strongest signal intensity of differential metabolites. During the progression from sepsis to S‐AKI, the overlap between differentially expressed proteins and metabolites across different functional nephron segments was limited. Hypergeometric tests confirmed that the observed overlaps were significantly lower than expected by chance for both DEMs and DEPs in all pairwise comparisons between compartments (*p* < 0.05), highlighting marked functional heterogeneity in injury mechanisms among renal compartments (Figures 1D and S1A).

**FIGURE 2:**
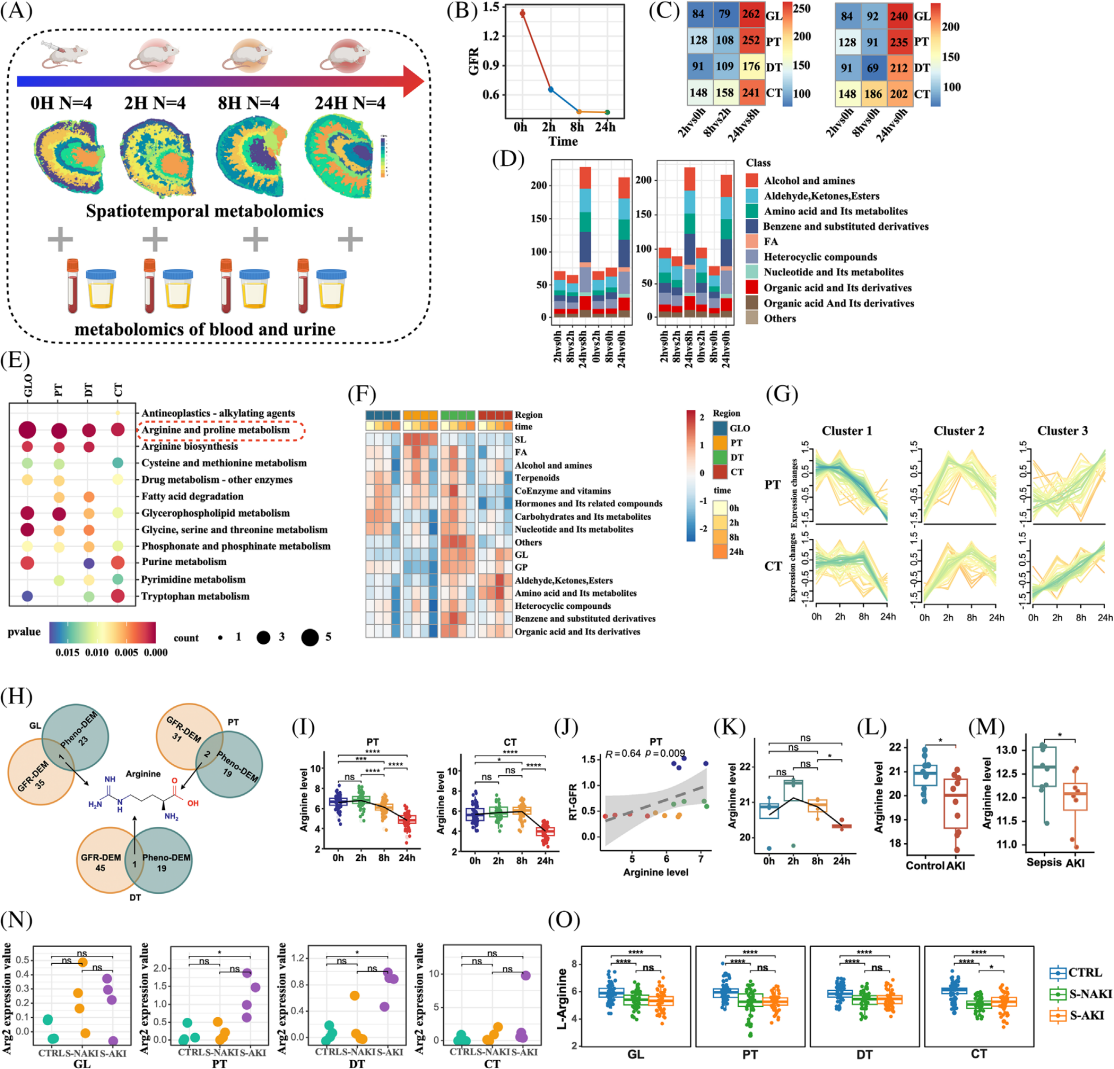
Spatiotemporal metabolomics reveals dynamic metabolic landscape during AKI progression. Mice kidney tissue sections and blood samples were collected at different time points for metabolomic profiling. (B) GFR at different time points during the progression of AKI. (C) Heatmap showing the number of DEMs between different time points. (D) Bar chart displays the number of DEMs belonging to different biochemical classes. (E) KEGG pathway enrichment of DEMs. (F) Heatmap depicts the average expression levels for each category of DEMs across all time points. (G) Three distinct clusters of metabolites identified by time‑series analysis. (H) Venn diagram shows the intersection between DEMs correlated with phenotype and DEMs correlated with GFR. (I) Arginine levels in kidney tissue over time. (J) Correlation between renal arginine levels and corresponding GFR. (K) Plasma arginine levels over time. (L) Arginine levels in urine samples of control mice and AKI mice. (M) Arginine levels in urine samples of sepsis patients and AKI patients. (N) Expression levels of ARG2 in four renal compartments across groups. (O) L‐Arginine levels in four renal compartments across groups.

Integrated enrichment analysis of differential proteins and metabolites further confirmed this regional specificity of injury functions (Figures 1E and S1B). Analysis focused on the most severely affected regions, the collecting duct and proximal tubule, revealed that the perturbed metabolic processes were primarily associated with amino acid metabolism, lipid metabolism and energy metabolism. Notably, dysregulation of arginine biosynthesis was a consistently observed phenomenon across all four renal compartments examined, suggesting a potentially pivotal role for arginine metabolism in the pathogenesis of AKI. Independent enrichment analysis of the differential proteins showed that the most significantly altered biological processes were related to immune activation, energy metabolism, and nucleotide metabolism (Figures 1F,G, and S2A,B). In addition, we observed a significant downregulation of arginine levels in S‐AKI, accompanied by markedly increased expression of arginase 2 (ARG2), which is consistent with arginine catabolism observed in the metabolic pathway alterations (Figure 2N,O).

To identify key metabolites and proteins significantly associated with the progression from the normal state to sepsis and subsequently to S‐AKI, as well as to explore their potential as biomarkers, we employed the LASSO method to construct machine learning (ML) classification models for each of the four functional renal compartments. The importance of differential metabolites and proteins was ranked in each model (Figure 1H). The constructed ML models showed strong fit to S‐AKI progression, particularly in the proximal tubule and collecting duct. In the proximal tubule, the mean squared error (MSE) was .005 for the DEP‐based model and .063 for the DEM‐based model. In the collecting duct, the MSE values were .001 for the DEP‐based model and .137 for the DEM‐based model, respectively (Figures 1I and S3A,B).

The relatively higher MSE observed in DEM‐based models compared with DEP‐based models may be attributable to the greater susceptibility of metabolite levels to physiological fluctuations, which may compromise the stability of metabolite‐based predictions. The relative importance of metabolites and proteins within the ML models is visualized in the bubble plot (Figure 1J, S3C).

### 2.2 Temporal Dynamics of Renal Metabolic Alterations and Arginine Decline in S‐AKI Progression

To precisely delineate the spatiotemporal dynamics of renal metabolites during the development and progression of S‐AKI, we conducted a longitudinal study by collecting kidney tissue and blood samples from mice at four critical time points (0, 2, 8 and 24 h) post‐S‐AKI induction. Simultaneously, we continuously monitored real time glomerular filtration rate (RT‐GFR) as a key functional readout (Figure 2A). The data revealed a time‐dependent, progressive decline in RT‐GFR (Figure 2B), confirming the successful establishment of a dynamic S‐AKI mice model characterized by deteriorating renal function. We then performed spatial metabolomics profiling on kidney tissues collected at these different time points. Pairwise comparative analysis between time points revealed that the number of significantly DEMs progressively increased as time advanced (Figure 2C). These findings indicate a progressive worsening of metabolic dysregulation and expanding tissue damage as S‐AKI advanced.

These DEMs encompassed a diverse chemical class, including amino acids, fatty acids, nucleotides, alcohols, amines, aldehydes, ketones, esters, organic acids and their derivatives, underscoring the systemic disruption of the renal metabolic network in S‐AKI (Figures 2D and S4A). Amino acids and fatty acids constituted the largest proportions, suggesting significant alterations in energy and lipid metabolism patterns. Changes in nucleotides and their related metabolites further suggest that nucleic acid synthesis and degradation are affected, which consequently alters the states of cellular proliferation and apoptosis. Subsequent pathway enrichment analysis of the DEMs identified prominent enrichment in multiple amino acid metabolism pathways, with arginine and proline metabolism and arginine biosynthesis being the most prominently affected. Significant pathway perturbations extended to amino acid metabolism (glycine‐serine‐threonine, tryptophan, cysteine‐methionine), fatty acid β‐oxidation, nucleotide turnover and glycerophospholipid remodelling (Figure 2E).

The majority of DEMs exhibited progressive downregulation during S‐AKI progression, reflecting an overall suppression of renal metabolic function and energetic failure. In contrast, levels of aldehydes, ketones, esters, amino acids, organic acids, benzenes and heterocyclic compounds were upregulated specifically in the collecting duct (Figure 2F). This regional heterogeneity indicates that the proximal and distal tubules are more susceptible to early damage, leading to metabolic disturbances and a decrease in the levels of related metabolites. In contrast, the collecting ducts are relatively resistant and become activated to perform compensatory functions. As a result, their repair mechanisms, stress response, and energy metabolism activities are enhanced, leading to an increased expression of metabolites such as aldehydes, ketones, esters, amino acids and organic acids. To systematically characterize the temporal expression patterns of DEMs during S‐AKI progression, we performed unsupervised clustering analysis for time‐series data, which identified three distinct patterns: a decreasing type (cluster 1), acute peak type (cluster 2), and increasing type (cluster 3) (Figures 2G and S4B). Cluster 1 (decreasing type) was predominantly composed of amino acids and their metabolites (e.g., arginine, N,N‐dimethylarginine) and benzene derivatives, suggesting suppression of amino acid metabolism. Cluster 2 (acute peak type) was enriched in organic acids and glycerophospholipids (e.g., L‐homoarginine, palmitoyl‐L‐carnitine), reflecting acute metabolic stress. Cluster 3 (increasing type) was dominated by glycerophospholipids and heterocyclic compounds (e.g., creatinine, LysoPE species), suggesting involvement in repair and compensatory processes. To identify metabolites directly associated with renal functional impairment, we computed correlations between DEM levels and RT‐GFR, further refining our list to RT‐GFR‐associated metabolites.

Spatial metabolomics data from three pathophysiological states (Control, Sepsis, S‐AKI) identified metabolites associated with the progression of S‐AKI. Subsequent spatiotemporal metabolomic analysis further explored key metabolites during the acute phase of AKI. By intersecting the DEMs derived from both spatial and temporal analyses, we found that arginine was significantly dysregulated across three renal compartments (Figure 2H). Notably, arginine levels exhibited a continuous decline during S‐AKI progression and showed a significant positive correlation with GFR (Figures 2I,J and S4C,D). In addition, arginine levels were significantly downregulated in the urine and serum of S‐AKI mice (Figure 2K,L). To assess the clinical translatability of this finding, we established a human cohort of sepsis and S‐AKI patients, confirming a significant reduction of arginine in the S‐AKI urine samples (*p* < 0.05) (Figure 2 M).

Integrating these findings with our prior functional analyses, which confirmed dysregulation of the arginine metabolic pathway (Figure 1E), we conclude that arginine is a pivotal molecule critically implicated in the progression of S‐AKI. Its consistent downregulation across spatial compartments, temporal stages, species (mice and human) and biofluids (serum and urine), coupled with its strong correlation with renal function (RT‐GFR), positions it not merely as a biomarker but as a central player in the underlying pathophysiology of S‐AKI.

### 2.3 ARG2 expression is increased in S‐AKI mice and arginase inhibitor effectively suppressed sepsis acute kidney injury

**FIGURE 3:**
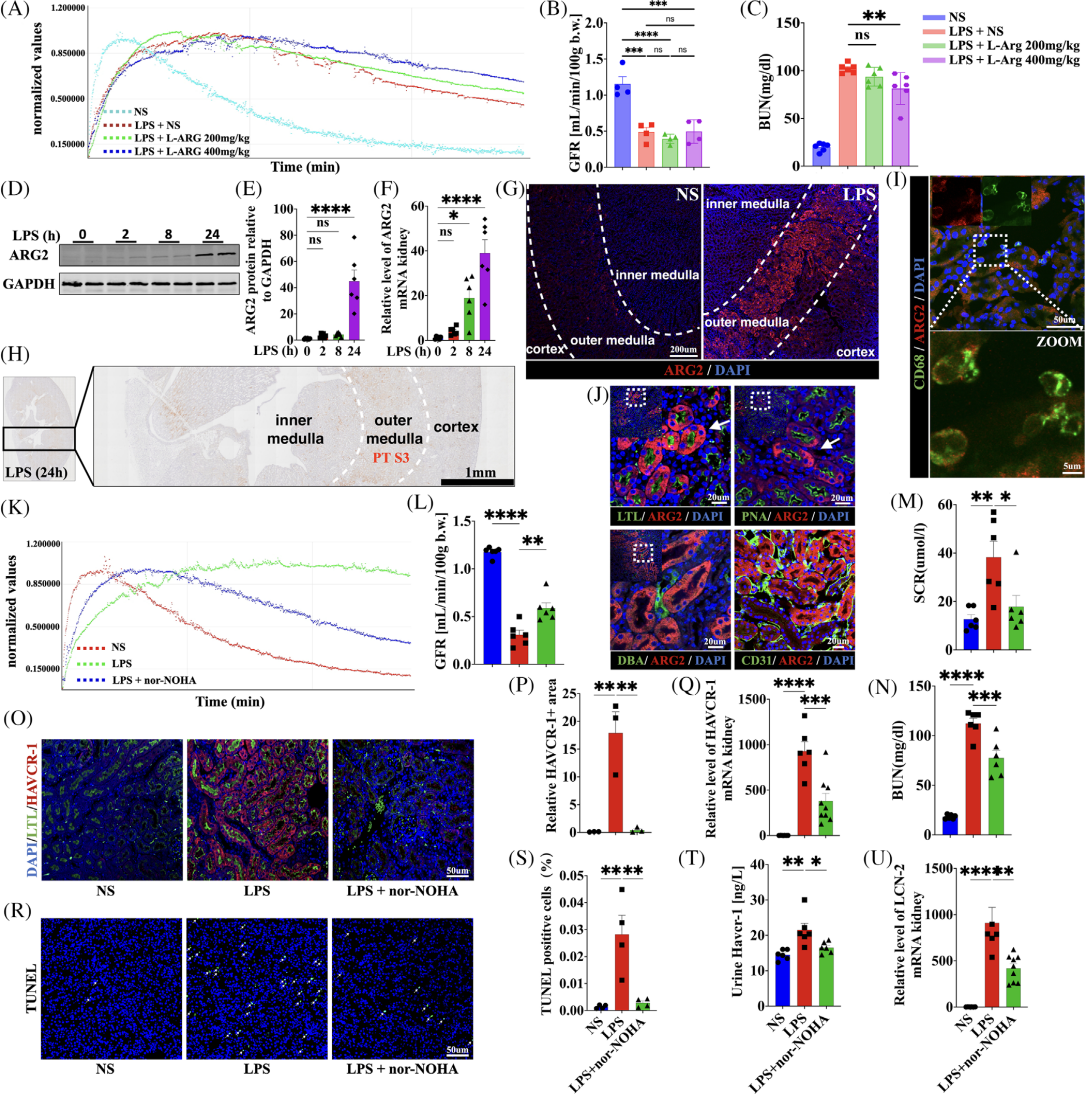
ARG2 expression is increased in S‐AKI mice and arginase inhibitor effectively suppressed sepsis acute kidney injury. (A) Representative images of real‐time glomerular filtration rate FITC excretion curves of S‐AKI mice intravenously supplemented with L‐arginine. (B) Real‐time glomerular filtration rate levels in S‐AKI mice intravenously supplemented with low and high doses of L‐arginine (*N* = 4). (C) Serum urea nitrogen levels in S‐AKI mice intravenously supplemented with low and high doses of L‐arginine (*n *= 6). (D and E) Western blot analysis (D) and densitometric quantification (E) of ARG2 protein levels in renal tissues from mice on hours 0–24 after LPS treatment (*n* = 6). (F) RT‐qPCR analysis of ARG2 mRNA expression in renal tissues from mice on hours 0–24 after LPS treatment (*n* = 6). (G) Representative immunofluorescence images showing co‐staining of ARG2 (red) with DAPI (blue) in renal sections from mice on hours 24 after LPS treatment. (H) Representative immunohistochemical staining images showing co‐staining of ARG2 (red) with DAPI (blue) in renal sections from mice on hours 24 after LPS treatment. (I) Representative immunofluorescence images showing co‐staining of ARG2 (red) with CD68 (green) in renal sections from mice on hours 24 after LPS treatment. (J) Representative immunofluorescence images showing co‐staining of ARG2 (red) with CD31, PNA, LTL and DBA (green) in renal sections from mice on hours 24 after LPS treatment. (K, L) Real time glomerular filtration rate from NS or nor‐NOHA treated mice after LPS treatment (*n* = 6). (M, N) Serum creatinine and urea nitrogen levels in NS or nor‐NOHA treated mice after LPS treatment (*n *= 6). (O, P) Representative immunofluorescence images show Havcr1 (red) co‐staining with LTL (green) and their fluorescent expression levels in kidney sections from NS or nor‐NOHA treated mice after LPS treatment (*n *= 3). (Q) RT‐qPCR analysis of Havcr1 mRNA expression in renal tissues from NS or nor‐NOHA treated mice after LPS treatment (*n* = 6–9). (R, S) Representative TUNEL staining images show Havcr1 (red) co‐staining with DAPI (blue) in kidney sections from LPS treated NS or nor‐NOHA treated mice, as well as their proportion of positive cell numbers (*n* = 3). (T) Urinary Havcr1 levels in NS or nor‐NOHA treated mice after LPS treatment (*n* = 6). (U) RT‐qPCR analysis of LCN2 mRNA expression in renal tissues from NS or nor‐NOHA treated mice after LPS treatment (*n *= 6–9). Data were expressed as means ± SEM. Statistically differences were determined using one‐way ANOVA followed by the Tukey's post hoc test. *p < .05, **p < .01, ***p < .001, ****p < .0001.

The role of L‐arginine supplements in patients with sepsis remains unclear.^18^ Drawing on previous study,^19^ we administered low and high concentrations of L‐arginine intravenously to S‐AKI mice. Although a tail vein injection of 400 mg/kg L‐arginine slightly reduced serum urea nitrogen (BUN) levels, no significant improvement in RT‐GFR was observed (Figure 3A–C). The kidneys, as the primary site for L‐arginine synthesis and transport, express renal arginase (ARG2), which is involved in the catabolism of renal L‐arginine and is highly expressed in various acute kidney injury models. However, the localization and mechanisms of ARG2 expression in the kidneys during AKI remain controversial. In our study, we found that ARG2 expression progressively increased over time during S‐AKI (Figure 3D–F). Further immunofluorescence and immunohistochemistry confirmed its predominant expression in the renal outer medulla (Figure 3G,H). ARG2 displays ubiquitous tissue expression and is inducible by LPS, TNFα and hypoxic stress.^20^ Contrary to previous research, we found that during S‐AKI, ARG2 was mainly localized in renal proximal tubules, distal tubules and macrophages, but not in endothelial cells or collecting ducts (Figure 3I,J). In contrast agent and ischemia reperfusion related renal injury, ARG2 appears to be expressed in the collecting ducts but not in macrophages.^10^, ^11^

Next, we treated the mice with the arginase inhibitor Nω‐hydroxy‐nor‐L‐arginine (nor‐NOHA). The results showed that nor‐NOHA significantly improved the decrease in RT‐GFR (Figure 3K,L), reduced serum creatinine (CRE) and BUN levels (Figure 3 M,N), and also lowered the levels of HAVCR1 in the kidneys and urine, as well as LCN2 mRNA levels in the kidneys (Figure 3O–Q,T,U). It also alleviated renal apoptosis (Figure 3R,S). In summary, arginase inhibitors may alleviate S‐AKI by inhibiting ARG2.

### 2.4 Arginase inhibitor nor‐NOHA alleviate S‐AKI by activating PPARγ to alleviate renal tubular lipid accumulation

**FIGURE 4:**
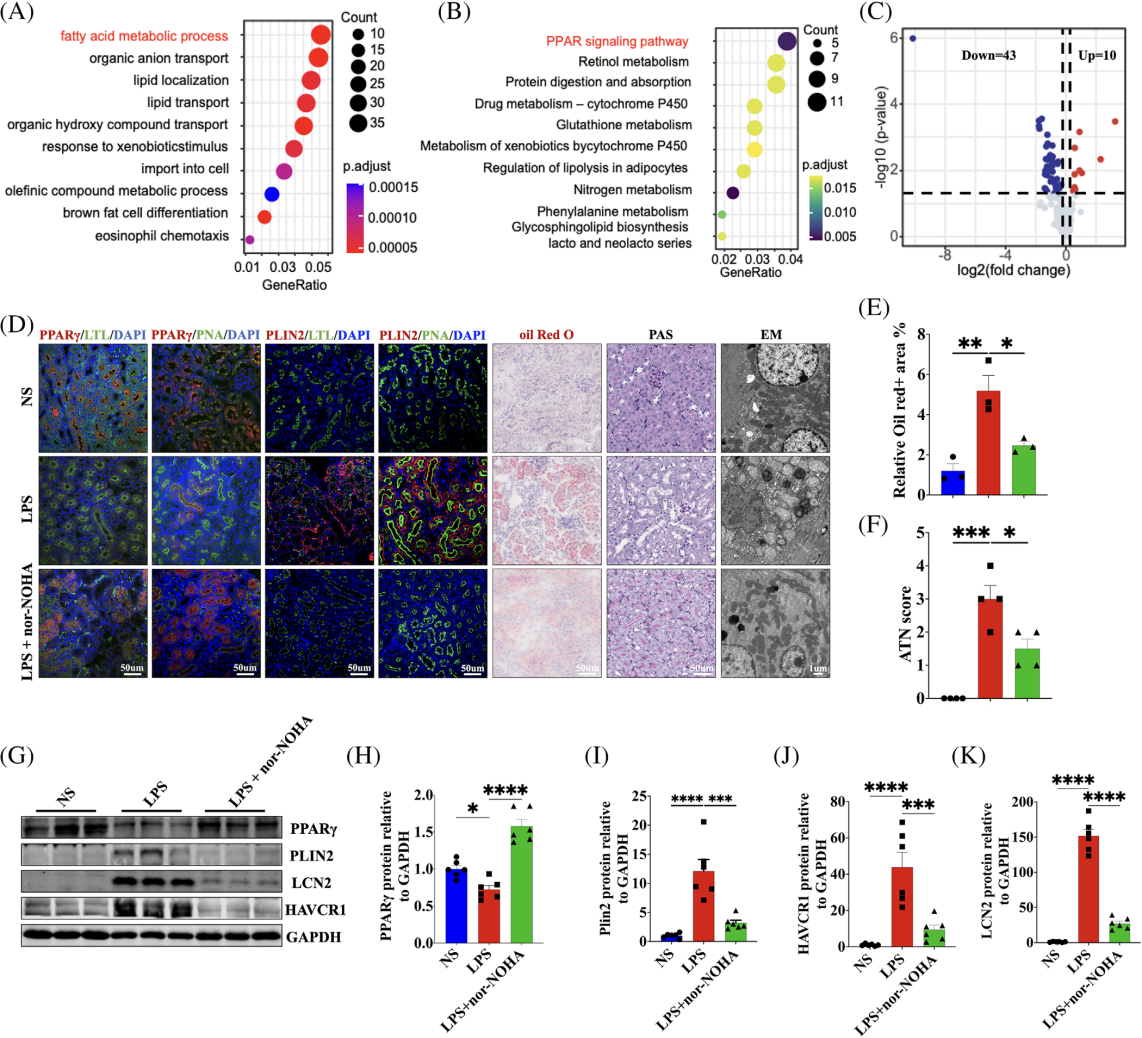
Arginase inhibitor Nω‐hydroxy‐nor‐L‐arginine (nor‐NOHA) alleviate sepsis‐induced acute kidney injury by activating PPARγ to alleviates renal tubular lipid accumulation. (A) GO analysis of the significantly differential genes between S‐AKI mice and nor‐NOHA‐treated mice (*n *= 6 mice/group). (B) KEGG analysis of the significantly differential genes between S‐AKI mice and nor‐NOHA‐treated mice (*n *= 6 mice/group). (C) Volcano plot of serum lipid metabolomics after treatment of S‐AKI with nor‐NOHA (*n *= 6–10 mice/group). (D) Immunofluorescence (red: PPARγ; PLIN2, green: LTL; PNA, blue: DAPI), glycogen staining, and electron microscopy representative images of NS and nor‐NOHA treated S‐AKI groups. (E) Quantification of oil red O staining intensity in NS, S‐AKI, and nor‐NOHA treated S‐AKI groups (*n* = 3). (F) Histologic damage score was measured in 5 randomly chosen fields per kidney using PAS‐stained kidney section (*n* = 4). (G, H, I, J, K) Western blot analysis and densitometric quantification of PPARγ, PLIN2, HAVCR1 and LCN2 protein levels in renal tissues from NS and nor‐NOHA treated S‐AKI groups (*n* = 6). Data were expressed as means ± SEM. Statistically differences were determined using one‐way ANOVA followed by the Tukey's post hoc test. *p < .05, **p < .01, ***p < .001, ****p < .0001.

To explore the potential mechanism by which nor‐NOHA alleviates S‐AKI, RNA‐seq analysis of renal tissues was combined with metabolomic analysis of serum. The principal component analysis results showed obvious differences among the healthy control group, the S‐AKI group with nor‐NOHA intervention, and the S‐AKI group without nor‐NOHA intervention (Figure S5A). nor‐NOHA intervention led to the upregulation of 578 genes and the downregulation of 118 genes (Figure S5B). The Gene Ontology (GO) enrichment analysis results indicated that the fatty acid metabolism pathway was significantly enriched. Renal tissue relies predominantly on FAO for energy. AKI‐mediated FAO impairment triggers energy depletion and lipid‐induced toxicity, driving tubular cell injury and mortality.^21^ The KEGG enrichment analysis results of differentially expressed genes showed that the PPAR pathway had the most significant changes (Figure 4A,B). The PPAR pathway plays an important role in AKI, mainly through regulating fatty acid metabolism, anti‐inflammatory, antioxidant, and anti‐apoptotic mechanisms to exert protective effects.^7^ It is divided into the following subtypes: PPARα, PPARβ/δ and PPARγ. The RNA‐seq results indicated that PPARγ had the most significant changes (Figure S5C). Meanwhile, the serum metabolomic results showed a significant decrease in serum lipid levels (Figure 4C). Therefore, we speculate that nor‐NOHA may alleviate tubular lipid accumulation during S‐AKI by affecting the expression of PPARγ.

To verify the above hypothesis, we first validated the results in mice. The results showed that there was obvious lipid accumulation in renal tubules during S‐AKI, including the expression of PLIN2 (lipid droplet coating protein) at the outer edge of the proximal and distal tubules, as well as Oil Red O staining results that confirmed significant lipid accumulation in renal tubules. At the same time, downregulated expression of PPARγ was observed in the proximal and distal tubules. However, nor‐NOHA intervention significantly reversed the above results, improved the degree of renal tubular injury and mitochondrial swelling (Figure 4D–F), and also improved the protein levels of renal injury markers LCN2, HAVCR1 and PLIN2 (Figure 4G–K). Studies have shown that lipid accumulation during AKI is closely related to the progression of late renal fibrosis.^8^ Therefore, we established a model of late renal fibrosis caused by sepsis.^22^ The results showed that nor‐NOHA significantly alleviated renal fibrosis levels on Day 15 (Figure S6A–F). In summary, nor‐NOHA may alleviate S‐AKI by inhibiting ARG2, thereby reversing the downregulation of PPARγ.

### 2.5 Renal tubular ARG2 knockdown mitigates lipid accumulation and injury in S‐AKI

**FIGURE 5:**
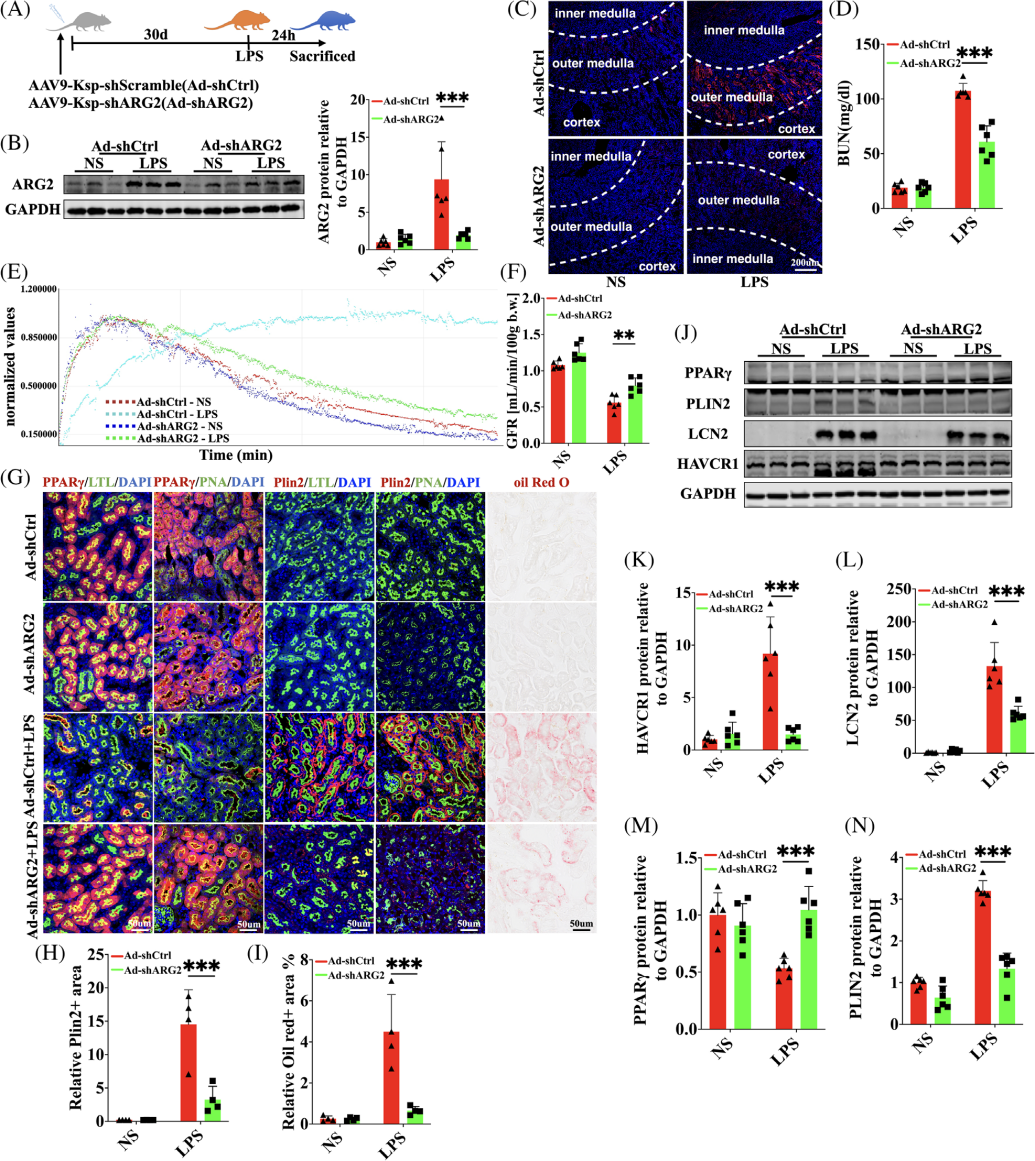
Renal tubular ARG2 knockdown mitigates lipid accumulation and injury in S‐AKI. Schematic illustration of the experimental workflow. Mice were i.v. injected with adeno‐associated virus serotype 9 (AAV9)‐Ksp promoter short hairpin ARG2 (AAV9. shARG2) or the control vector AAV9‐Ksp promoter‐shScramble (AAV9. shCtrl) for 30 days, then were treatment with LPS. (B) Western blot analysis and densitometric quantification of ARG2 protein levels in renal tissues from mice treated with AAV9.shCtrl or AAV9.shARG2 under the NS or LPS treatment (*n* = 6). (C) Representative images of ARG2 and DAPI staining in kidneys from mice treated with AAV9.shCtrl or AAV9.shARG2 under the NS or LPS treatment. (D) Serum urea nitrogen levels in mice treated with AAV9.shCtrl or AAV9.shARG2 under the NS or LPS treatment (*n* = 6). (E, F) Real time glomerular filtration rate from mice treated with AAV9.shCtrl or AAV9.shARG2 under the NS or LPS treatment (*n* = 6). (G–I) Immunofluorescence (red: PPARγ; PLIN2, green: LTL; PNA, blue: DAPI), representative images of mice treated with AAV9.shCtrl or AAV9.shARG2 under the NS or LPS treatment (*n* = 4). (J–N) Western blot analysis and densitometric quantification of PPARγ, PLIN2, HAVCR1 and LCN2 protein levels in renal tissues from mice treated with AAV9.shCtrl or AAV9.shARG2 under the NS or LPS treatment (*n* = 6). Data were expressed as means ± SEM. Statistically differences were determined using one‐way ANOVA followed by the Tukey's post hoc test. *p < .05, **p < .01, ***p < .001, ****p < .0001.

NOHA and nor‐NOHA show superior ARG2 inhibition (20× and 10× vs. ARG1) but lack isoform specificity, constraining their use.^23^ To rule out their effects on ARG1 and ARG2 in other cell types (hepatocytes, macrophages and neutrophils, etc.), we injected an adenovirus with a renal tubule‐specific promoter (KSP‐Cadherin) that knocks down ARG2 via the tail vein. The results showed that the expression of renal tubular ARG2 was significantly inhibited (Figure 5A–C). Knockdown of renal tubular ARG2 reduced BUN levels and increased RT‐GFR (Figure 5D–F). Knockdown of renal tubular ARG2 decreased the protein expression levels of renal injury markers HAVCR1 and LCN2, as well as the lipid droplet surface marker protein PLIN2, while increasing the protein expression level of PPARγ (Figure 5G–N). In summary, the above results indicate that ARG2 may mediate renal tubular lipid accumulation and renal injury through PPARγ.

### 2.6 ARG2 knockout alleviates tubular lipid accumulation and renal injury in vitro

**FIGURE 6:**
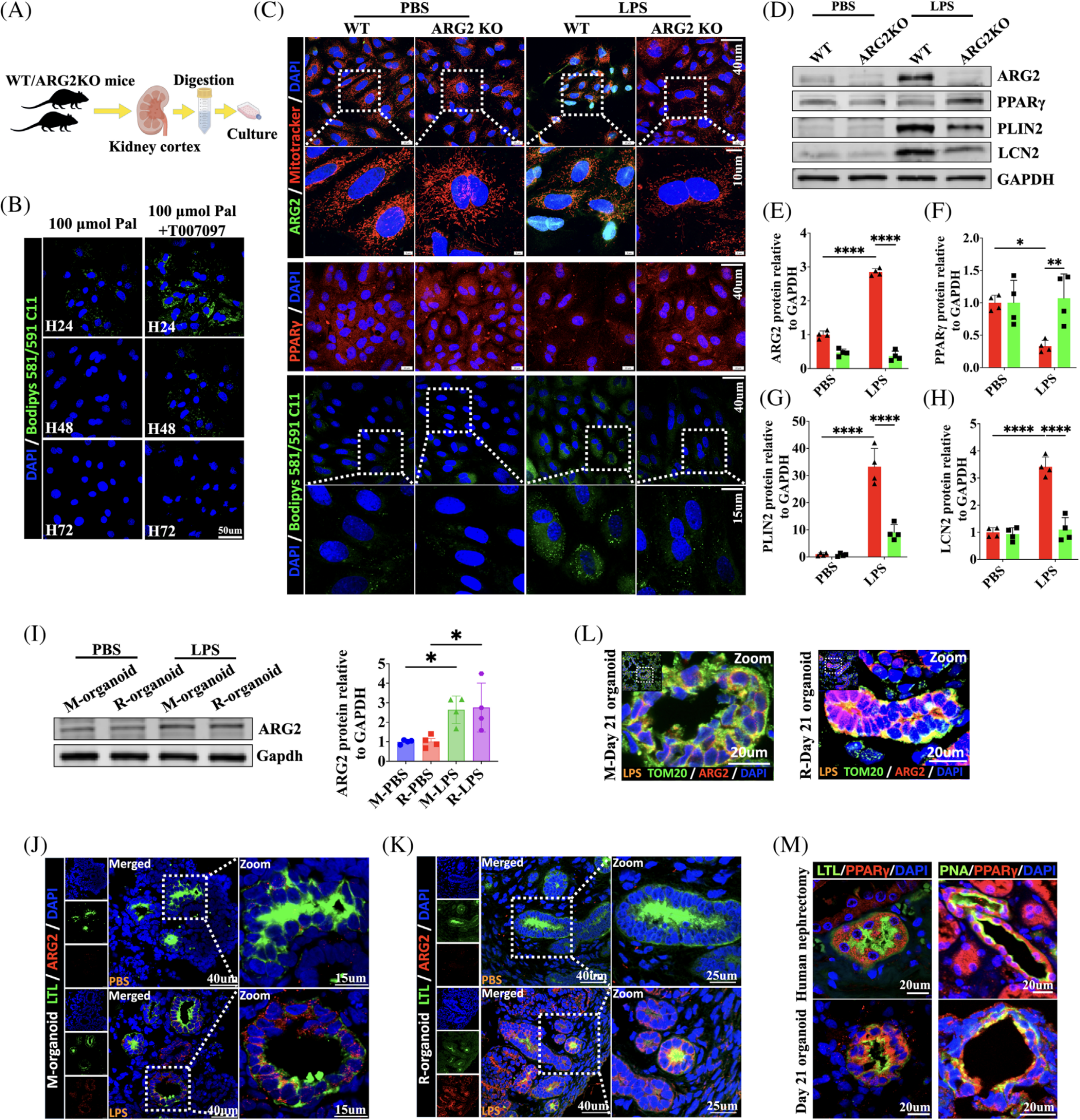
ARG2 knockout alleviates tubular lipid accumulation and renal injury in vitro. (A) Schematic illustration of the experimental workflow. (B) Immunofluorescence (green: Bodipys, blue: DAPI), representative images of primary renal tubular epithelial cells treated with palmitic acid and T7007097. (C) Representative immunofluorescence images of renal tubular epithelial cells isolated from WT and ARG2KO mice after LPS and PBS treatment (red: MitoTracker; PPARγ, green: ARG2; Bodipys, blue: DAPI). (D–H) Western blot analysis and densitometric quantification of ARG2 (E), PPARγ (F), PLIN2 (G) and LCN2 (H) protein levels in renal tubular epithelial cells isolated from WT and ARG2KO mice after LPS and PBS treatment (*n* = 4). (I) Western blot analysis and densitometric quantification of ARG2 protein levels in kidney organoids after LPS and PBS treatment (*n* = 4). (J, K) Representative immunofluorescence images of kidney organoids after LPS treatment (red: ARG2, green: LTL, blue: DAPI). (L) Representative immunofluorescence images of kidney organoids after LPS treatment (red: ARG2, green: TOM20, blue: DAPI). (M) Representative immunofluorescence images of kidney organoids after LPS treatment and human kidney tissue (red: ARG2, green: TOM20, blue: DAPI). Data were expressed as means ± SEM. Statistically differences were determined using one‐way ANOVA followed by the Tukey's post hoc test. *p < .05, **p < .01, ***p < .001, ****p < .0001.

In vitro, we isolated renal primary tubular epithelial cells (RPTCs) from the kidneys of wild‐type and global ARG2 knockout mice (Figure 6A). First, we treated the RPTCs with 100 µmol of palmitic acid (PAL) for 24 hours to simulate lipid accumulation, and set up groups treated with the PPARγ inhibitor (T007097) and control groups (PBS). The results showed that at 24 h, the lipid droplet levels in the RPTCs treated with T007097 were significantly higher than those in the control group, and at 48 h, there was still a certain degree of lipid droplet accumulation, while the lipid droplets in the control group were completely cleared at 48 h (Figure 6B). This indicates that PPARγ plays a crucial role in lipid droplet metabolism. Next, we treated the RPTCs with LPS. The results showed that ARG2 is located in the mitochondria of RPTCs. Moreover, knockout of ARG2 alleviated the decrease in PPARγ, reduced lipid droplet accumulation in RPTCs, and decreased the expression of injury markers (Figure 6C–H).

Poor concordance between animal and human trial outcomes limits preclinical translation. Human cell‐based tools offer a complementary approach to conventional animal models in drug development. To this end, we referred to the differentiation protocols of Dr. Morizane and Dr. Ryuichi's team and constructed human embryonic stem cell‐derived kidney organoids. After treatment with LPS, ARG2 expression was significantly increased (Figure 6I) and mainly localized in the renal tubules (Figure 6J,K). Further co‐staining with the mitochondrial outer membrane marker TOM20 revealed that ARG2 is located on the mitochondrial outer membrane of renal tubules (Figure 6L). We performed immunofluorescence staining for PPARγ, and the results showed that PPARγ has the same expression pattern as in adult kidneys (Figure 6 M).

### 2.7 ARG2 knockout alleviates kidney organoids lipid accumulation and renal injury

**FIGURE 7:**
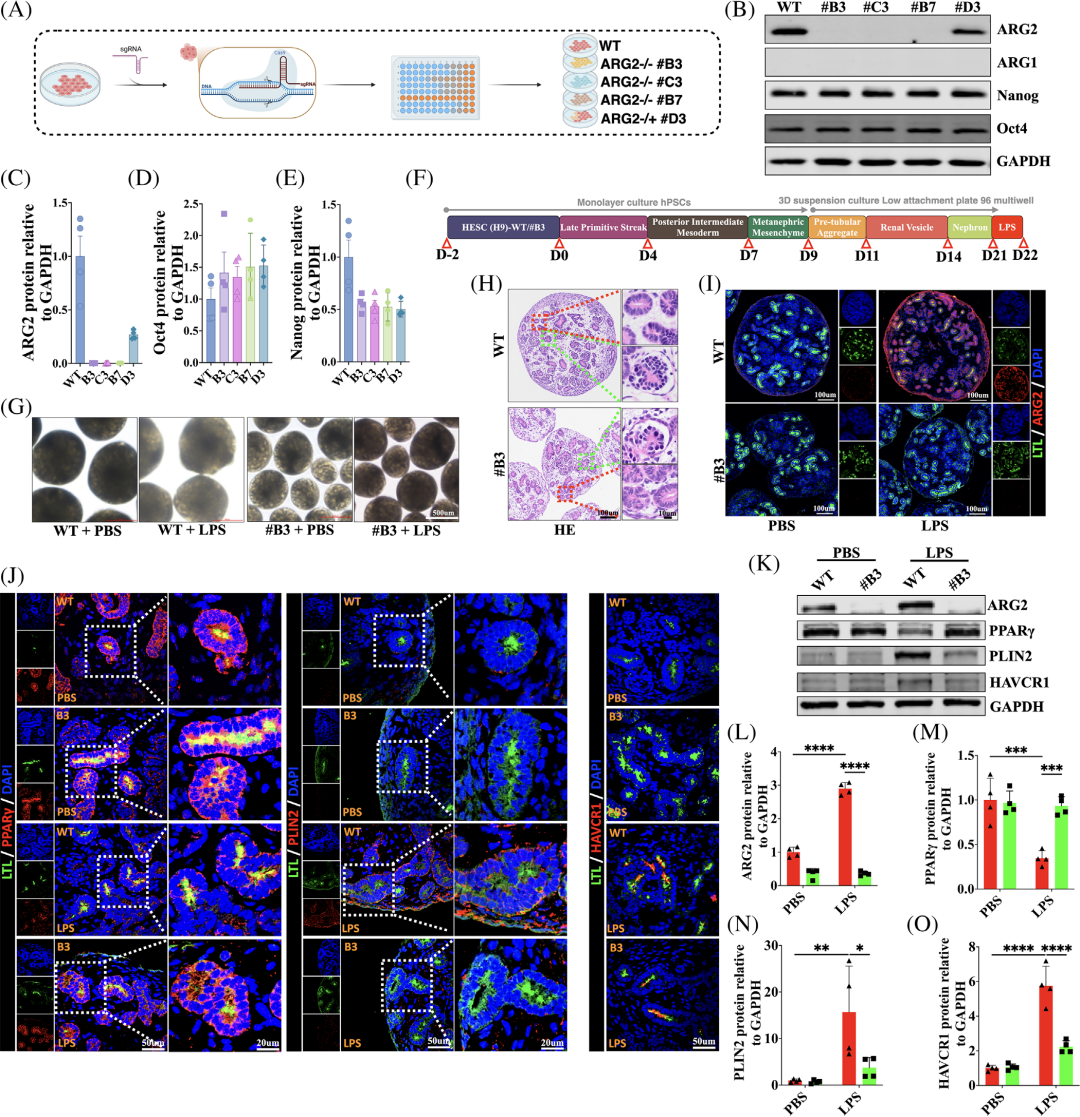
ARG2 knockout alleviates kidney organoids lipid accumulation and renal injury. (A) Flowchart of constructing the ARG2KO human embryonic stem cell line using the CRISPR‐Cas9 technology. (B–E) Western blot analysis and densitometric quantification of ARG2, ARG1 NANOG and OCT4 protein levels from human embryonic stem cell line (*n *= 4). (F) Flowchart of kidney organoid construction process. (G) Representative bright‐field images of WT and ARG2KO embryonic stem cell‐derived kidney organoids after treatment with LPS and PBS. (H) Representative images of kidney organoids derived from WT and ARG2KO embryonic stem cells after treatment with LPS and PBS using HE‐staining. (I) Representative immunofluorescence images of WT and ARG2KO embryonic stem cell‐derived kidney organoids after LPS and PBS treatment (red: ARG2, green: LTL, blue: DAPI). (J) Representative immunofluorescence images of WT and ARG2KO embryonic stem cell‐derived kidney organoids after LPS and PBS treatment (red: PPARγ; PLIN2; HAVCR1, green: LTL, blue: DAPI). (K–O) Western blot analysis and densitometric quantification of ARG2, PPARγ PLIN2 and HAVCR1 protein levels of WT and ARG2KO embryonic stem cell‐derived kidney organoids after LPS and PBS treatment (*n *= 4). Data were expressed as means ± SEM. Statistically differences were determined using one‐way ANOVA followed by the Tukey's post hoc test. *p < .05, **p < .01, ***p < .001, ****p < .0001.

We constructed an ARG2KO human embryonic stem cell line (HESC, H9) based on CRISPR‐Cas9 technology (Figure 7A). EC predominantly expresses ARG2, the extrahepatic isoform, which fuels polyamine synthesis via ornithine production to modulate cell proliferation and differentiation.^24^ Therefore, we tested the differentiation ability of the ARG2 knockout embryonic stem cell line. The results showed that the knockout of ARG2 did not affect its differentiation ability (including the expression levels of OCT4 and NANOG), but we did not detect the expression of ARG1 protein (Figure 7B–E). Next, we differentiated WT and ARG2 knockout homozygotes into kidney organoids (Figure 7F). From the bright‐field and HE‐staining results, it can be seen that the knockout of ARG2 did not significantly affect the differentiation of kidney organoids, which have distinct glomerular and tubular structures (Figure 7G,H).

Next, we treated WT and ARG2KO kidney organoids with LPS and PBS, respectively. The results showed that the knockout of ARG2 restored the expression levels of PPARγ in renal tubules, alleviated the levels of lipid droplet markers in renal tubules, and inhibited the levels of renal injury marker HAVCR1 (Figure 7I–O).

## 3 DISCUSSION

Despite significant advances in understanding the pathophysiology and diagnostic markers of S‐AKI, it remains a major clinical issue and burden, and further study is needed to reduce the acute and chronic consequences.^4^ Macrophages, vascular ECs and TECs represent key cellular mediators in the pathophysiology of S‐AKI, for which treatment options are predominantly supportive.

Glucose, lipid and amino acid metabolic reprogramming enables cells to acquire nutrients and energy for adapting to environmental challenges and danger cues.^25^, ^26^, ^27^ In the studies of renal spatial metabolomics and plasma metabolomics, we found that during S‐AKI, the content of L‐arginine in the kidneys and plasma showed a significant downward trend over time. As is well known, L‐arginine depletion often occurs during sepsis.^28^, ^29^, ^30^ The kidneys, as the primary site for regulating circulating L‐arginine levels, synthesize L‐arginine from citrulline absorbed by the gut.^31^, ^32^ Although the liver also synthesizes L‐arginine, the L‐arginine it produces is mainly used for the urea cycle. Therefore, renal L‐arginine metabolism during sepsis may be the main cause of L‐arginine depletion. However, the therapeutic effects of L‐arginine supplementation in sepsis patients are still controversial.^33^ In our study, it was found that L‐arginine supplementation seems not to alter the severity of S‐AKI. In mammals, arginase plays a key role in the urea cycle in dealing with toxic ammonia, catalysing the conversion of L‐arginine to L‐ornithine and urea.^34^ Arginase I (ARG1) and ARG2 are two isoforms of arginase.^35^ The ARG1 is primarily expressed in the liver and is expressed at low levels in the bone marrow, while the ARG2 gene is present in almost all tissues (kidney, prostate, gastrointestinal tract, muscle, and endocrine tissues).^36^ ARG2 is primarily localized in the mitochondria and is highly expressed in the kidneys,^37^ However, the physiological role it plays in the kidneys remains unknown.

In various types of AKI, the role of ARG2 is highly controversial. In ischemia‐reperfusion acute kidney injury, ARG2 is mainly upregulated in the renal cortex of mice, where it exacerbates renal injury by mediating nitrosative stress in renal tubular epithelial cells. Renal function is improved following ARG2 genetic deficiency and nor‐NOHA intervention.^11^ However, other studies have shown that ARG2 plays an important role in the formation of urea and osmotic gradients in the cortex and medulla. The upregulation of ARG2 has been shown to alleviate the extent of renal injury in ischemia‐reperfusion injury.^14^ In the cisplatin‐induced nephrotoxicity model, studies have shown that ARG2 is primarily expressed in macrophages rather than in renal tubular cells. In mice with global genetic deficiency of ARG2, there is a reduction in renal inflammatory macrophage infiltration and a decrease in the secretion of interleukin‐6 and interleukin‐1β. The results indicate that ARG2 promotes the inflammatory response of macrophages through mitochondrial reactive oxygen species.^12^ Contrary to previous studies, in the S‐AKI model, we found that ARG2 was mainly located in the outer medullary proximal and distal parts and macrophage types in the kidney. Although we constructed ARG2 ‐knockout mice, its role in immune cells (macrophages) seems to be significant.^38^ We found that it exacerbated the degree of renal injury, which was largely attributed to the massive infiltration of pro‐inflammatory macrophages (Figure S7A–E). To rule this out, we used tail vein injection of adenovirus with renal tubule‐specific promoter (KSP) to knock down ARG2 expression in renal tubular cells. The study results indicated that ARG2 may regulate the expression of PPARγ.

PPARγ, a nuclear receptor‐transcription factor hybrid, upregulates perilipin‐mediated FA transport and storage. Studies have shown that PPARγ exerts a protective effect in AKI by promoting the fatty acid β‐oxidation pathway.^9^, ^39^ In the kidneys of high‐fat diet‐induced obese mice, the expression and activity of ARG2 are significantly enhanced, with renal lipid accumulation, increased ROS levels, and elevated renal injury markers. In high‐fat diet‐fed ARG2 knockout mice, the degree of renal injury and lipid accumulation is significantly reduced, indicating that ARG2 plays an important role in the development of obesity‐related nephropathy.^17^ In adipocytes, the absence of ARG2 can enhance the activation of AMPK‐α. Activated AMPK‐α can induce the expression and activity of PGC‐1α. PGC‐1α can coordinate the functions of PPARγ and PPARα, promoting mitochondrial biogenesis and function. This includes the expression of enzymes involved in mitochondrial FAO and oxidative phosphorylation. This in turn protects PPARγ expression and helps maintain the normal metabolic functions of adipocytes.^16^ Therefore, we propose that during S‐AKI, ARG2 may regulate the expression of PPARγ, thereby alleviating renal tubular lipid accumulation and the extent of renal injury.

HPSC‐derived kidney organoids now enable modelling of renal development, drug‐induced nephrotoxicity and kidney disease.^40^, ^41^ We constructed ARG2KO embryonic stem cells based on CRISPR‐Cas9 technology and differentiated them into kidney organoids. We simulated an in vitro sepsis‐induced acute kidney injury model by treating the kidney organoids with LPS and confirmed the above hypothesis in the kidney organoids.

There are some limitations of this study. First, the protective role of ARG2 in S‐AKI has been primarily investigated in cellular and mouse models, rather than in human samples. Although human kidney organoid models can well simulate the development and function of renal tubules, they lack the vascularization and immune system components present in in vivo models, and thus cannot reproduce the interactions between immune cells and renal tubule cells in the kidneys. Second, although it is known that the activation of ARG2 affects lipid metabolism by regulating PPARγ, thereby exacerbating S‐AKI, the regulatory mechanisms between ARG2 and PPARγ have not been thoroughly explored. Third, although nor‐NOHA exhibits higher affinity for ARG2, the potential confounding effects resulting from ARG1 inhibition cannot be completely excluded. The future development of ARG2‐specific inhibitors is therefore essential for elucidating the pathophysiological roles of ARG2 and advancing targeted therapeutic strategies,

In conclusion, suppressing ARG2 expression in renal tubules during S‐AKI may mitigate renal tubular lipid accumulation and kidney injury via the PPARγ, offering a novel potential therapeutic approach for S‐AKI treatment.

## 4 METHODS

Additional details for methods are provided in the Supporting Information (Methods).

### 4.1 Establishment and treatment of septic acute kidney injury model

Male C57BL/6 mice aged 8–12 weeks were purchased from Charles River Laboratories. C57BL/6_ARG2^(‐/‐)^ mice aged 8–12 weeks were obtained from Cyagen (Figure S8). The animals were housed individually under a 12‐h light/dark cycle and had free access to food and water. They were acclimated for 7–10 days prior to study initiation with institutional ethical approval.

S‐AKI was induced by a single intraperitoneal injection of LPS (Sigma, #L4130) at a dose of 10 mg/kg. Sepsis‐associated chronic kidney injury (S‐CKD) was established by intraperitoneal injection of LPS (1 mg/kg) every other day until the 15th day.^42^ Nω‐hydroxy‐nor‐L‐arginine (nor‐NOHA, Bachem, #4027934) was dissolved in PBS at a concentration of 10 mg/mL. A single intraperitoneal injection of nor‐NOHA (50 mg/kg body weight) was administered to C57BL/6 mice 30 min before LPS injection.^11^

### 4.2 Percutaneous real‐time glomerular filtration rate monitoring

RT‐GFR was assessed as described.^43^, ^44^ After depilation and isoflurane induction, the MediBeacon device was placed on the flank. Baseline recording, FITC‐sinistrin i.v. injection, and ∼1 h monitoring followed; data were analysed in MB Studio v.22.

### 4.3 Induction of kidney organoids and treatments

Kidney organoids were generated by Morizane protocol,^40^ made a slight modification. HESCs were differentiated via sequential CHIR99021 (8 µM, Day 0), Activin A (10 ng/mL, Day 4), and FGF9 (10 ng/mL, Day 7) treatment in Advanced RPMI 1640. Following Accutase dissociation (Day 9), 1 × 10^5^ cells/well were seeded in CHIR (3 µM)/FGF9 (20 ng/mL) onto 96‐well ultra‐low‐attachment plates, with medium replacement on days 11 (FGF9) and 14 (growth factor‐free). Organoids were cultured at 37°C/5% CO_2_ with triweekly 100 µL medium exchanges for ≥7 days.

Kidney organoids were generated by Ryuichi's protocol,^45^ made a slight modification. HESCs were dissociated with Accutase and seeded at 1 × 10^4^ cells/well in U‐bottom low‐attachment 96‐well plates. Aggregates were cultured in basal differentiation medium (DMEM/F12, B27, ITS, NEAA, β‐ME, GlutaMAX, penicillin‐streptomycin) supplemented with Y‐27632 (10 µM), Activin A (1 ng/mL), and bFGF (20 ng/mL; R&D Systems, #3718‐FB‐025). After 24 h, embryoid bodies (EBs) were transferred to fresh plates and maintained for 6 days in CHIR99021 (10 µM)/Y‐27632 (10 µM), with half‐medium exchanges every 2 days. On Day 7, EBs were switched to Activin A (10 ng/mL)/BMP4 (3 ng/mL; #314‐BP)/CHIR (3 µM)/RA (0.1 µM; Selleck, #S2924)/Y‐27632 (10 µM) for 3 days without medium change. From Days 10–13, organoids were cultured in CHIR (1 µM)/FGF9 (5 ng/mL)/Y‐27632 (10 µM). On day 14, organoids were transferred to transwell inserts in KR medium (DMEM/F12, GlutaMAX, NEAA, diluted β‐ME, penicillin‐streptomycin, KSR; Gibco, #10828028) with FGF9 (10 ng/mL)/CHIR (3 µM) for 2 days. From Day 16, KR medium was refreshed every 48 h until maturation (Days 21–23).

### 4.4 Statistical analysis

Results are presented as mean ± SD. Intergroup differences were evaluated using ANOVA (one‐, two‐, or three‐way) followed by Tukey's multiple comparisons test. Analysis was performed in GraphPad Prism 9.4.1, with *p* < 0.05 considered statistically significant.

## AUTHOR CONTRIBUTIONS

Hongxue Meng, Xubin Zheng, Lixin Cheng, Kaijiang Yu and Changsong Wang conceived the study, designed and performed the experiments, and contributed to the writing of the manuscript. Yu Xin, Yanqi Liu, Ning Zhang, Pengfei Huang and Qianqian Zhang participated in the experiments and data analysis. Yinghao Luo, Xinran Wang, Xiuhua Zhang, Tao Wu, Fengye Liu and Yu Xiao assisted with the animal studies. Lifeng Shen, Weiting Zhang, Xibo Wang and Xianglin Meng contributed to the cell‐based studies. Yuping Bai, Xuan Wang, Nan Zhang and Qianghong Xu provided technical support for the study. All authors contributed to the article and approved the submitted version. All the authors are responsible for the content of the manuscript.

## CONFLICT OF INTEREST STATEMENT

The authors declare no conflicts of interest.

## ETHICAL APPROVAL

The clinical investigation was approved by the Ethics Committee of the Second Affiliated Hospital of Harbin Medical University (Ethical Approval Number: KY2021‐188) and strictly complied with the ethical standards of the Declaration of Helsinki. In this study, all research was conducted in accordance with the Declaration of Helsinki. Written informed consent was duly obtained from all patients.

## Supporting information



Supporting Information: ctm270770‐sup‐0001‐SuppMat.zip

## Data Availability

All the data supporting the conclusions presented in this article are included in this published article. All data and analysis methods are described in the article. Renal metabolomics, renal proteomics, serum metabolomics, and targeted quantitative serum metabolomics data are available in the Supplemental materials.
