# Supplementary material for: Arginase 2 deficiency mitigates sepsis‐associated acute kidney injury by alleviating lipid accumulation
Source: Clin Transl Med. 2026 Sep 18;16(9):e70770. doi: 10.1002/ctm2.70770 (PMC13588275; doi:10.1002/ctm2.70770)
Supplement: Supplementary file 1 [file CTM2-16-e70770-s001.zip › ctm270770-sup-0009-TableS1.docx]

**Table S1**

| **Antibody/Fluorescein** | | **Manufacturer/item No** | **dilution ratio** |
| --- | --- | --- | --- |
| **Primary antibodies** | | | |
| ARG2 | | CST, #55003 | IF/IHC, 1:100; WB: 1:1000 |
| PPARγ | | Proteintech, #16643-1-AP | IF, 1:200; WB: 1:1000 |
| PLIN2 | | Proteintech, #15294-1-AP | IF, 1:500; WB: 1:1000 |
| HAVCR1 | | Proteintech, #30948-1-AP | IF, 1:100; WB: 1:1000 |
| HAVCR1 | | R&D, #AF1750 | IF, 1:100; WB: 1:1000 |
| CD68 | | Thermo, #14-0681-95 | IF, 1:100 |
| LCN2 | | Proteintech, #26991-1-AP | WB, 1:1000 |
| GAPDH | | Abcam, #ab8245 | WB, 1:5000 |
| TOM20 | | Proteintech, #66777-1-Ig | IF, 1:100 |
| αSMA | | Thermo, #MA5-41117 | IF, 1:100 |
| VIM | | Thermo, #PA5-27231 | IF, 1:100 |
| NANOG | | Proteintech, #14295-1-AP | IF, 1:100; WB: 1:1000 |
| OCT4 | | Proteintech, #11263-1-AP | IF, 1:100; WB: 1:1000 |
| CD31 | | R&D, #AF3628 | IF, 1:100 |
| INOS | | Abcam, #ab178945 | IF, 1:100 |
| F4/80 | | Abcam, #ab6640 | IF, 1:100 |
| **Second antibodies** | | | |
| Alexa Fluor™ Plus 488 Anti-Rabbit IgG | | Abcam, #150077 | IF, 1:250 |
| Alexa Fluor™ Plus 555 Anti-Rat IgG | | Abcam, #221240 | IF, 1:250 |
| Alexa Fluor™ Plus 647 Anti-Rabbit IgG | | Abcam, #150075 | IF, 1:250 |
| Alexa Fluor™ Plus 488 Anti-Mouse IgG | | Abcam, #150113 | IF, 1:250 |
| Alexa Fluor™ Plus 647 Anti-Goat IgG | | Abcam, #150135 | IF, 1:250 |
| Alexa Fluor™ Plus 647 Anti-Rat IgG | | Abcam, #150159 | IF, 1:250 |
| Alexa Fluor™ Plus 647 Anti-Mouse IgG | | Abcam, #150115 | IF, 1:250 |
| **Fluorescein** | | | |
| Bodipys 581/591 C11 | | Byontime, #C2053S | / |
| Mitotracker_555 | | Absin, #47038879 | / |
| LTL_FITC | | Vrctorlab, #FL-1321-2 | IF, 1:100 |
| PNA_FITC | | Vrctorlab, #FL-1071 | IF, 1:250 |
| DBA_FITC | | Vrctorlab, #FL-1031 | IF, 1:50 |
| **Primer** | | | |
| **Species** | **Gene** | **Forward Primer** | **Reverse Primer** |
| **Mouse** | Arg2 | ACGGGCAAATTCCTTGCGT | TGGACCATATTCCACTCCTAGC |
|  | Havcr1 | GTCTGTATTGTTGTCGAGTGGAG | CGTGTGGGAATCTCTGGTTTAAC |
|  | Lcn2 | GGACCAGGGCTGTCGCTACT | GGTGGCCACTTGCACATTGT |
|  | Gapdh | ACTCCACTCACGGCAAATTC | TCTCCATGGTGGTGAAGACA |
